# Natriuretic Peptide Testing in Primary Care

**DOI:** 10.2174/157340308786349499

**Published:** 2008-11

**Authors:** Shafiq U Rehman, James L Januzzi

**Affiliations:** Department of Medicine and Division of Cardiology, Harvard Medical School and Massachusetts General Hospital, Boston, MA, 02114, USA

## Abstract

The incidence, as well as the morbidity and mortality associated with heart failure (HF) continue to rise despite advances in diagnostics and therapeutics. A recent advance in the diagnostic and therapeutic approach to HF is the use of natriuretic peptide (NP) testing, including both B-type natriuretic peptide (BNP) and its amino terminal cleavage equivalent (NT-proBNP). NPs may be elevated at an early stage among those with symptoms as well among those without. The optimal approach for applying NP testing in *general populations *is to select the target population and optimal cut off values carefully. Superior diagnostic performance is observed among those with higher baseline risk (such as hypertensives or diabetics). As well, unlike for acute HF, the cut off value for outpatient testing for BNP is 20-40 pg/mL and for NTproBNP it is 100-150 ng/L. In *symptomatic *primary care patients, both BNP and NT-proBNP serve as excellent tools for excluding HF based on their excellent negative predictive values and their use may be cost effective. Among those with established HF, it is logical to assume that titration of treatment to achieve lower NPs levels may be advantageous. There are several ongoing trials looking at that prospect.

## INTRODUCTION

Heart failure (HF) is a major and growing public health problem in the United States. The prevalence of HF in US is estimated around 5 million, and over 550 000 new cases are diagnosed with HF each year [1]. HF is the primary reason for 12 to 15 million office visits and 6.5million hospital days each year. The incidence, as well as the morbidity and mortality associated with HF continues to rise despite advances in diagnostics and therapeutics [2]; this is in part due to the fact that ’salvage’ of patients with myocardial infarction is in part more successful, but also reflects the age-related increase in the incidence of risk factors for HF, such as hypertension. In addition, there is an equally large pool of patients with myocardial dysfunction but without apparent HF symptoms, or at worst, minor symptoms. This further complicates decision making, revealing inadequacy of clinical means alone to recognize HF [3]. 

In addition to the diagnostic challenges, the timing and sequence of initiating treatments, gauging adequacy of therapy and monitoring of therapy for adverse events are significant challenges. Due to these factors the current care of patients with HF remains suboptimal [4] and many HF patients are hospitalized routinely for reasons that can be averted with good outpatient care [5]

A recent advance in the diagnostic and therapeutic approach to HF is the use of natriuretic peptide testing. Including both B-type natriuretic peptide (BNP) and its amino terminal cleavage equivalent (NT-proBNP), this class of biomarker has proven to be more useful than original expectations. This review will discuss recent advances in the use of natriuretic peptide testing to assist in the primary care recognition and management of patients with suspected or proven HF.

### Heart Failure as a Progressive Disorder

HF is a progressive disorder accompanied with changes in left ventricular (LV) structure and geometry such that the chamber dilates and/or hypertrophies and becomes more spherical—a process referred to as cardiac remodeling. This change further deteriorates myocardial function which in turn perpetuates the remodeling process. Cardiac remodeling generally precedes the development of symptoms (occasionally by months or even years) and may continue despite treatment. 

Several factors can accelerate the process of LV remodeling, of these the activation of endogenous neurohormonal systems plays a vital role in cardiac remodeling and thereby in the progression of HF. Because natriuretic peptides (NPs) are produced in the heart, they may serve as a biological marker of LV remodeling and changes in circulating NPs may parallel the changes in LV structure [6-8].

## BIOLOGY OF BNP AND NT-proBNP

### Synthesis, Release and Physiological Actions

Human BNP is synthesized as a pre-pro-peptide which is cleaved by endoprotease yielding a 108-amino-acid pro-peptide (proBNP1-108) [9]. The propeptide is further cleaved by a furin- or corin-like endoprotease [10] to form biologically active BNP and inactive N-terminal proBNP. Both of these peptides circulate in human plasma [11].

Multiple mechanisms induce the BNP gene, however, mechanical stretch is probably the most potent inducer of the BNP gene expression in cardiac myocytes [12]. In addition to myocytes, cardiac fibroblasts and the coronary vasculature have also been shown to express the BNP gene [13,14]. While BNP mRNA concentrations are higher in the atria than the ventricles [9], given the greater ventricular mass, total content of BNP mRNA is greater in the ventricle [15]; thus the ventricles are the main determinants of serum concentration of BNP under both physiologic and pathological conditions [16,17]. Besides stretch, additional stimuli for BNP release have been proposed including endotoxemia [18], hypoxia [19], and ischemia [20].

While BNP may have several endocrine functions including vasodilation, natriuresis, inhibition of the sympathetic nervous as well as the renin-angiotensin aldosterone system [21], it may also exhibits some autocrine and paracrine functions, including regulation of myocyte growth, inhibition of fibroblast proliferation and extracellular matrix deposition, a cytoprotective anti-ischemic function, and influences on coronary endothelium and vascular smooth muscle proliferation and contractility [20, 22]. NT-proBNP, on the other hand is considered biologically inert [23], but its elevation nonetheless reflects activity of the BNP gene, and NT-proBNP appears to provide comparable information to BNP in most circumstances.

### Circulating Concentrations of Natriuretic Peptides

Plasma concentrations of both NPs (BNP and NT-proBNP) in healthy persons are much lower than in diseased states, are quite comparable to each other (unlike in disease states), and are more modified by physiologic factors including age, gender and drugs than in the disease state.

NPs increase with age [24], probably related to the more frequent occurrence of systolic, and diastolic dysfunction and ventricular hypertrophy in the elderly, as well as the drop in renal function (requisite for clearance of both peptides) seen with age [25]; NPs are higher in women [24], possibly due to estrogen levels in women in reproductive age [26] or effects of androgens in men, leading to lower values in males [27]. Circadian variation may be seen in BNP/NT-proBNP concentrations, although these levels are small and clinically insignificant [28]. 

The effects of drugs on circulating NP concentrations are complex. Cardiovascular drugs with favorable effects on the neurohormonal system such as diuretics, angiotensin-converting enzyme inhibitors, β-adrenergic blockers, and spironolactone may lower NP values, in parallel with the improved prognosis seen with treatment with these drugs [29-52].

### Biological Variability

The biological variation of NPs secretion must be considered when interpreting serial natriuretic peptide results. Biological variability refers to the expected day-to-day or week-to-week changes in NPs levels measured in apparently stable patients in response to physiologic changes such as changes vascular tone and filling pressures, without overt destabilization [53-55], as well as possibly changes in clearance as well [56]. In addition, analytical imprecision and alternate splicing of BNP and/or NT-proBNP may also contribute to variability in circulating levels [57, 58].

Recent studies suggest that in stable chronic heart failure patients, long-term intra-individual variation in natriuretic peptide levels is in the order of 30%, with a change of more than 23% likely to indicate a clinically significant change beyond background variation [59, 60]. NT-proBNP appears much less subject to wide biologic variability and appears relatively constant with variability that may be less than 10% [59]. 

In summary, considering biological variation, changes in BNP or NT-proBNP in the range of 25-30% likely reflects a “true” change, and may be interpretable in the context of therapeutic monitoring. While NT-proBNP appears less subject to the effects of biological variability than BNP, the significance of this finding relative to the value for management of HF patients remains unclear.

Despite biological variability and conditions that modify NP levels, NPs remain potentially useful to discern a wide range of clinical conditions in HF patients. The applicability of natriuretic peptides is well accepted in inpatient care both in an emergency department setting as well as in a hospitalized patient with respect to diagnosis and prognosis, moreover, it has been found useful for screening, diagnosis and prognosis in outpatient care as well (Table **1**).

## CLINICAL APPLICATIONS OF NATRIURETIC PEPTIDES IN PRIMARY CARE

### Screening for Asymptomatic Ventricular Dysfunction 

The widespread use of echocardiography for screening purposes in general population is limited by its costs and lack of availability in primary care settings. NPs levels may provide an important tool in screening asymptomatic patients with LV dysfunction as NPs may be elevated in these patients from conditions that predispose to clinical HF including myocardial ischemia, inflammation, fibrosis, hypertrophy, and valvulopathy [19-22,65,66,]. The mechanisms of NPs elevation in these conditions at pre-clinical stage before accumulation of fluid and stretch of myocardium are thought to be related to their potential role in modulation of ventricular structure/remodeling/fibrosis. [22,65-67]. As it has been proposed that their synthesis is transcriptionaly activated in response to the activation of rennin-angiotensin system and other neurohormonal pathways involved in heart failure

Screening for asymptomatic LV dysfunction is particularly important as prevalence of asymptomatic LV systolic dysfunction alone is estimated from 0.9% to 12.9% [62], depending on baseline risk. Furthermore, among asymptomatic patients early detection of cardiac remodeling or related structural and functional impairment may allow preventive therapies to be initiated earlier delaying progression to HF [63, 64]. Moreover outcome data suggests that among general population, people with modest elevations in NPs are at increased risk for developing cardiovascular morbidity and mortality [68-70]. However, for accurate screening of sub clinical disease a clear delineation of the optimal cut off values and understanding of major cardiac (ischemia, arrhythmia) and non cardiac (age, gender, obesity, anemia, renal function) sources of variation in NPs in the population is necessary. The natriuretic peptide cut points used in primary care are considerably lower than for emergency department-based evaluation of acute dyspnea, and use of these higher cut points would be expected to result in reduced sensitivity of BNP or NT-proBNP. Indeed, as demonstrated by Tang and colleagues [61], the use of a BNP cut point of 100 pg/mL was associated with a disastrously low sensitivity among symptomatic HF patients in a HF/transplant clinic.

Among asymptomatic individuals in the community, NPs have been investigated for their ability to detect increased LV mass and impaired systolic and, or diastolic dysfunction. However, their performance relies on the characteristics determined by patient population. In a low risk population, lower pretest probability render NPs suboptimal for general screening of population [71-74], in addition, screening with NP’s may be cost prohibitive. In a study by Rosenfield *et al.*, [71] BNP testing was found to be sub-optimal for screening patients with pre clinical systolic or diastolic dysfunction. Comparable results were reported in the Framingham Offspring Study by Vasan *et al.,* [74], who observed suboptimal diagnostic accuracy of NPs for detection of mild left ventricular dysfunction (LVSD) (EF<50%) and LV mass especially among women. However, the diagnostic performance improved in women when moderate LSVD was targeted (EF <40%). 

In contrast, use of NPs to screen select populations with higher risk for developing HF (including patients with coronary artery disease, hypertension, or diabetes mellitus who do not yet demonstrate impaired LV function, hypertrophy, or geometric chamber distortion, so called AHA stage A) may be advantageous.

It has been shown in population studies examining such patients that NP levels are generally higher in diabetics, hypertensives as well as those with increased LV mass [75]. These higher levels while prognostically relevant [76,77], may also be useful to identify those in whom targeted therapeutic intervention might be most appropriate.

Thus, depending upon the population investigated, there have been varying reports on the value of BNP for detecting LVSD in general populations (Table **2**). Areas under the receiver operating characteristic (ROC) curve have ranged from 0.59 [78] to 0.95 [79], suggesting a potential utility in higher-risk, targeted groups, to identify cardiac abnormalities and thus trigger further work up and more intensive treatment. 

From a perspective of modality of use, it is generally felt that the value of NP’s for widespread population screening is related to their negative predictive value (NPV), rather than their positive predictive value (PPV). That is, these assays are best used to exclude risk than to identify it. As an example, Epshteyn *et al.* [80] showed that in high risk asymptomatic diabetic population, BNP levels <39 pg/ml showed a high negative predictive value of 91%. In order to determine an appropriate BNP level which would preclude the need for echocardiography, it was shown that the cutoff point of 20 pg/mL had an impressive negative predictive value for those with systolic dysfunction (96%) or systolic plus diastolic dysfunction (100%) [81]. Similarly, for NT-proBNP a cut-off value of 125 pg/mL had the sensitivity, specificity, positive predictive value, and negative predictive values of 0.97, 0.46, 0.15 and 0.99, respectively to detect LVSD (EF < 40). Area under the ROC curve was 0.87. The application of an age-differentiated cut-off value for NT-proBNP (125 pg/mL for <75 years old and 450 pg/mL for > 75 years old) did not increase diagnostic performance [82]. 

Utility of BNP testing for identifying any structural heart disease in addition to LV systolic dysfunction was assessed in another study [83] involving general population. In ROC analyses, the AUC was suboptimal (0.77; 95% CI=0.74-0.79) for general screening of structural heart diseases including mild left ventricular systolic dysfunction, valvular heart disease, hypertensive heart disease, hypertrophic cardiomyopathy , ischemic heart disease and lone atrial fibrillation. This efficacy was improved when select groups with a high prevalence of heart disease were considered. For instance, those older then 65 (men, AUC = 0.88; women, AUC = 0.83) as well as those with hypertension or diabetes (men, AUC = 0.85; women, AUC = 0.83). Similar results were reported earlier in another Japanese study where a plasma BNP level of 40 pg/ml had a sensitivity of 85% and a specificity of 92% for heart disease detection. AUC for BNP was significant 0.94 and a plasma BNP level of 13 pg/ml or less had a 100% NPV for heart disease [84]. 

**Fig. (1):**
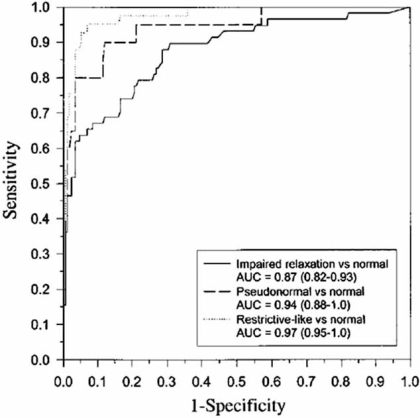
ROC curve comparing sensitivity and specificity of BNP and echocardiographic diagnosis of various diastolic filling patterns. Patients with abnormal systolic function were excluded. AUC is significant (P<0.001) for each diastolic filling abnormality vs. patients with normal systolic function (patients with other diastolic filling abnormalities excluded in each analysis). Lubien E *et al*. [86].

In patients with isolated diastolic dysfunction, NPs may be elevated and may reflect the severity of dysfunction [85-89]. Moreover, BNP and NT-proBNP are elevated in those with mild diastolic dysfunction and correlate with increased filling pressures during exercise [90,91]. In a study by Lubien *et al.* [86], the area under the ROC curve for BNP to detect any diastolic dysfunction was 0.92 (0.87–0.95; *p*<0.001); patients with restrictive-like filling patterns on echocardiography had the highest BNP levels (408±66 pg/mL) and AUC =0.97 (Fig. **1**). Importantly, however, while NP levels are unable to differentiate systolic *vs*. diastolic dysfunction [89] but in patients with symptoms of HF and normal systolic function, NP values may useful to predict diastolic abnormalities. 

Importantly, even in the absence of overt HF, modest elevations in plasma NPs levels are prognostically meaningful in population-based testing. In a population-based prospective study of 1991 subjects without apparent heart failure both BNP and NT-proBNP were predictive of mortality after a median follow up of 5.6 years even after adjustment for clinical and echocardiographic abnormalities including left ventricular hypertrophy and diastolic dysfunction [92]; in this analysis, NT-proBNP was clearly superior to BNP for prognostication. In another prospective study of 3346 persons without heart failure, elevated BNP values above the 80th percentile (20 pg/mL for men and 23 pg/mL for women) were associated with multivariable-adjusted hazard ratios of 1.62 for death (P=0.02), 1.76 for a first major cardiovascular event (P=0.03), 1.91 for atrial fibrillation (P= 0.02), 1.99 for stroke or transient ischemic attack (P=0.02), and 3.07 for heart failure (P=0.002) [40]. Similarly, in another study of general population, McDonagh *et al., *showed that BNP was an independent predictor of 4-year all-cause mortality [93]. 

Overall, studies would thus suggest that the optimal approach for applying NP testing to detect HF or prognosticate in general populations is to select the target population carefully, with superior performance observed among patients with a higher baseline risk (such as hypertensives or diabetics). As well, unlike for acute HF, where higher decision limits are used, the cut off value for outpatient testing for BNP is 20-40 ng/mL and for NT-proBNP it is 100-150 ng/L. Values below these cut-points exclude the presence of cardiac abnormalities with a high NPV; values above these cut-points suggest further evaluation is necessary but do not absolutely guarantee the presence of cardiac structural abnormalities. 

### Diagnostic Evaluation in Symptomatic Patients

**Fig. (2):**
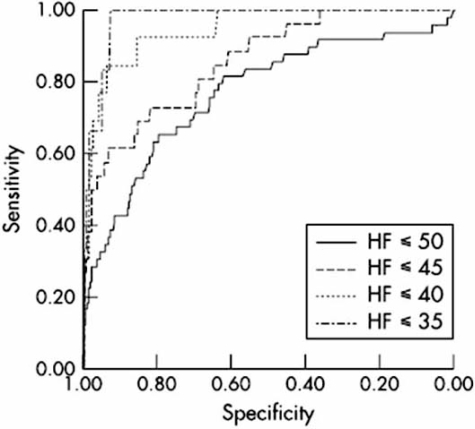
Receiver operating characteristic (ROC) curves for the ability of NT-proBNP to diagnose systolic HF and LVEF ≤50%/≤45%/≤ 40%/≤ 35%, respectively. For the diagnosis of heart failure ≤40, NT-proBNP = 106.7 pmol/l had sensitivity/specificity 0.92/0.86, positive predictive value /negative predictive value 0.11/1.00 and AUC = 0.94 Groenning BA *et al*. [96].

In symptomatic primary care patients, both BNP and NT-proBNP serve as excellent tools for excluding HF based on their excellent negative predictive values [94-100]. In patients with symptoms suggestive of HF, Cowie *et al.* [94] investigated the value of BNP and showed that a cut-off value of 22 pmol/L for BNP could rule out the diagnosis of HF with high NPV of 98%. Similarly, in a prospective, randomized controlled trial involving 305 patients with symptoms of dyspnea and/or peripheral edema, NT-proBNP measurement significantly improved the diagnostic accuracy by a general practitioner over and above clinical review by correctly ruling out heart failure [95]. In another study [96] (Fig. **2**), NT-proBNP levels identified those with symptoms of heart failure and LVEF ≤40% among general population with a sensitivity of 92%, a specificity of 86%, negative predictive values of 100% and area under the curve of 0.94. Similarly in another study [97] both BNP and NT-proBNP had excellent negative predictive values for exclusion of HF in patients with clinical suspicion of HF; in this study, a BNP at a cut-off of 40 pg/ml had a NPV of 88%, and an NT-proBNP of 150 pg/ml gave a NPV of 92%.

In a meta analysis involving 19 studies, 22 populations, authors suggested that in a low risk population appropriate use of NPs can help reduce the demands of echocardiogrpay and cardiac referrals [99]. In a recent study [101], NT-proBNP use among symptomatic patients in general practice was found useful for cost-effective exclusion of the diagnosis of heart failure (HF).

**Fig. (3):**
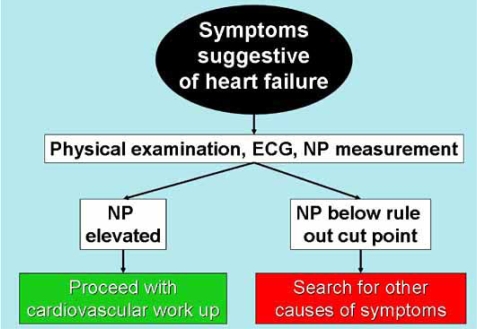
A suggested algorithm for evaluation of symptomatic patients.Clinical evaluation should be supplemented with natriuretic peptide measurement.

Thus, in the presence of symptoms, a general recommendation would be to apply NP testing with the goal to exclude HF. Using cut-points of 20-40 pg/mL for BNP and 125-150 ng/L for NT-proBNP provides excellent NPV in this regard. Results above these levels do not necessarily confirm HF, but suggest that further evaluation is necessary (Fig. **3**). Importantly, age exerts a significant effect on NP concentrations, thus, lower cut-points might be useful for younger patients, while higher cut-points (e.g. an NT-proBNP of 300-450 ng/L) might provide superior NPV for elders. The optimal cut-points for BNP in elders remain unclear.

### Outpatient Measurement of NPs in Patients with Established HF

The longitudinal clinical assessment of stability (and hence prognosis) in HF is difficult because of the lack of strong predictors of mortality or morbidity. Plasma natriuretic peptide levels increase in patients with symptomatic HF in proportion to the severity of disease and correlate with several indices of heart failure such as the New York Heart Association (NYHA) functional class, hemodynamics, left ventricular ejection fraction and filling pressure [102-104]. In patients with chronic HF, plasma BNP and NT-proBNP predict functional capacity and exercise tolerance [105-108]. In terms of mortality NPs predict mortality across the entire spectrum of HF patients, in those patients with advanced disease as well as those with minimally symptomatic left ventricular dysfunction [109,110]. In fact, among plasma levels of ANP, BNP, cGMP, and norepinephrine and clinical and hemodynamic parameters, only high levels of plasma BNP (P<.0001) and pulmonary capillary wedge pressure (P=.003) were significant independent predictors of the mortality in patients with HF by Cox proportional hazard analysis [109]. Accordingly, a recent systematic review suggested that BNP is a strong prognostic indicator at all stages of disease and seems to be a better predictor of survival than many traditional factors, such as NYHA class, serum creatinine, and even left ventricular ejection fraction [110]. Moreover, BNP and NT-proBNP, were independently related to 4-year mortality in patients with severe heart failure receiving established heart failure therapy including angiotensin-converting enzyme inhibitors and beta-blocker therapy [111]. 

While elevated baseline values of NPs are predictive of poorer outcomes, the prognostic value of the changing levels of BNP and NT-proBNP levels measured during treatment could be even more meaningful [112-115]. Furthermore, patients whose BNP values fail to fall in response to treatment tend to be at particularly high risk of death or a cardiovascular event [112,114,116,117]. Anand *et al. *[112] analyzed the significance of change in BNP over time from baseline to 4 and 12 months. While higher baseline values were predictive of morbidity and mortality, a further increase in BNP values over time led to further incremental rise in risk. On the other hand patients with the greatest percent decrease in BNP from baseline to 4 and 12 months had the lowest morbidity and mortality.

Because lower levels of BNP or NT-proBNP are associated with better clinical outcome, and patterns of NP concentrations appear to be more prognostic than single measurements, it is logical to assume that titration of treatment to achieve lower NPs levels may be advantageous. The logic behind this concept is that concentrations of NPs decrease following institution or intensification of several established therapies of HF; among these agents are diuretics, angiotensin converting enzyme (ACE) inhibitors, angiotensin receptor blockers (ARB), β blockers, spironolactone, and nesiritide [29-52]. 

While levels of BNP and NT-proBNP fall parallel to the fall in filling pressures with institution of diuretics and vasodilators [29-36], it is interesting to note that initiation of non-loop diuretics with relatively modest diuretic effects such as spironolactone may lead to substantial acute and chronic reductions in BNP and NT-proBNP [38-42]. The acute fall may be related to a diuretic effect followed by more significant reductions in BNP or NT-proBNP related to the suggested benefits of spironolactone on ventricular remodeling [43]. Similarly, ACE inhibitors besides decreasing filling pressures [32-36], in addition they may have an anti remodeling related effect on natriuretic peptide levels. The response to β-blockers is more complex. Introduction of metoprolol in stable mild HF is associated with an initial rise in NT-proBNP levels that is related to its secretion (or clearance) but is not due to clinical decompensation [44, 45]. Longer-term following β-blocker therapy initiation or adjustment, levels of natriuretic peptides fall, paralleling changes in LV remodeling [45, 46]. The response of NT-proBNP to carvedilol (and other vasodilator β-blockers) may be different from metoprolol, with an initial fall in natriuretic peptide levels [47-50]; since there were significant differences in the stages of HF of the studies comparing effects of β-blockers, these findings may be more due to different responses in NT-proBNP at different stages of HF, rather than heterogeneity in response to different agents. 

The clinical benefit of titrating therapy according to NP levels was tested in a landmark study by Troughton *et al.* [118] patients with decompensated HF and ejection fractions of <40% were recruited to test the hypothesis that any treatment that decreases NT-proBNP levels to <200 pmol/L (approximately 1700 ng/L) would reduce cardiac events. Their results showed that NT-proBNP guidance resulted in fewer combined heart failure decompensation, hospitalization and mortality events (19 versus 54, p=0.02). More recently, findings supportive of natriuretic peptide guided HF therapy in context of a more contemporary HF regimen were demonstrated in the recently published STARS-BNP study [119]. In this multicenter study, 220 patients with NYHA functional class II–IV heart failure were randomized to BNP and clinically guided treatment. Therapies adjusted to achieve a BNP (target value<100 ng/L) resulted in more frequent changes 134 versus 66 in clinical group (p < 0.05); with all types of HF drugs changed more frequently in the BNP group. After a median 15 months follow up, the primary composite end point (unplanned hospital stays for heart failure or death related to heart failure) was observed in 25 of 110 (24%) in the BNP group versus 57 of 110 (52%) in the clinical group, p < 0.001). Similarly, the STARBRITE study assessed the impact of targeting treatment to achieve an individualised BNP target during short-term follow-up [120]. While this strategy did not significantly reduce the number of days alive and out of hospital it did result in more optimal use of ACE inhibitor and β-blocker therapy, while diuretics were less likely to be increased. 

The effect of guiding treatment to achieve targeted levels of NPs is currently being tested in several larger randomised trials. Methods papers have been published for the BATTLESCARRED [121] and TIME-CHF [122] and many other studies are examining the role of BNP or NT-proBNP guidance for heart failure therapy. These studies will assess the effect of this strategy in large cohorts. 

## Figures and Tables

**Table 1.** Clinical Uses of Natriuretic Peptides.

Screening for HF in high risk populations
Prognostic evaluation in general population especially those with risk factor for heart disease.
Differentiation of HF from pulmonary conditions in dyspneic patients in primary care
Diagnosing HF in dyspneic patients in emergency department
Determining HF severity, Prognosis
Guiding HF therapy?

**Table 2.** Use of Natriuretic Peptides for Screening in Low Risk and High Risk Population

Cohort Type	Optimal Cut off Value	AUC	NPV	PPV

**Low Risk or General Population**				

Redfield *et al.*	25.9 pg/mL (BNP)	0.79	N/A	N/A
(EF ≤40% or moderate-to-severe diastolic dysfunction)				

Vasan *et al.*				
EF ≤50	45 pg/mL (BNP)	M=0.72	0.93	0.38
	50 pg/mL	W=0.56	0.98	0.07
EF≤40	51 pg/mL (BNP)	M=0.79	0.97	0.22
	50 pg/mL	W=0.85	1.00	0.04

**High Risk or Symptomatic**				

Gustafsson F *et al*.	125 pg/mL (NT-proBNP)	0.87	0.99	0.15
Systolic DysfunctionEF ≤0.40				

Faut *et al.*	40 pg/mL (BNP)	0.79	0.88	0.49
Systolic DysfunctionEF ≤0.40	150 pg/mL (NT-proBNP)	0.81	0.92	0.48

Krishnaswamy P *et al.*	48 pg/mL (BNP)	0.95	0.85	0.90
Systolic or Diastolic Dysfunction				

Lubien E *et al.*	62 pg/mL (BNP)	0.91	0.89	0.78
Diastolic Dysfunction					

Mak GS *et al.*	90 pg/mL (BNP)	0.89	0.98	0.36
Diastolic Dysfunction				
